# Machine learning-based prediction models for postoperative pulmonary complications in elderly patients undergoing abdominal surgery

**DOI:** 10.3389/fsurg.2026.1780650

**Published:** 2026-07-14

**Authors:** Qiang Zhong, Guiming Huang, Wen Zhou, Baolin Zhong, Yijian Chen, Jiegang Zhou, Junjun Li

**Affiliations:** Ganzhou Hospital-Nanfang Hospital, Southern Medical University (Ganzhou People’s Hospital), Ganzhou, Jiangxi, China

**Keywords:** abdominal surgery, elderly patients, machine learning, postoperative pulmonary complications, risk prediction, SHAP, XGBoost

## Abstract

**Background:**

Postoperative pulmonary complications (PPCs) are common adverse events after abdominal surgery in older adults, but existing risk scores may have limited transportability and clinical interpretability in elderly surgical populations.

**Methods:**

This retrospective cohort study included 2,456 patients aged >=65 years who underwent abdominal surgery in the development/internal cohort and 542 patients in an independent external-validation cohort. Six algorithms were compared, including logistic regression, random forest, support vector machine, neural network, XGBoost, and LightGBM. Model performance was evaluated using discrimination, calibration, decision curve analysis, and external validation. SHAP analysis was used to support interpretability. Additional revision analyses examined pulmonary-function-test missingness, minor versus major PPCs, PPC co-occurrence patterns, temporal stability, COVID-era effects, and comparator-score performance.

**Results:**

PPCs occurred in 425 of 2,456 patients (17.3%) in the development/internal cohort and 105 of 542 patients (19.4%) in the external-validation cohort. XGBoost showed the best overall performance, with AUCs of 0.856 (95% CI, 0.811–0.900) in the independent test set and 0.821 (95% CI, 0.781–0.861) in the external-validation cohort. At a 20% risk threshold, the independent-test sensitivity, specificity, PPV, and NPV were 87.5%, 60.9%, 32.0%, and 95.9%, respectively. SHAP analysis identified ASA physical status, COPD, upper abdominal surgery, age, emergency surgery, albumin, and surgical duration as leading contributors. The model separated patients into low-, moderate-, and high-risk groups with observed PPC rates of 6.9%, 24.4%, and 37.6%. Sensitivity analyses supported robustness to pulmonary-function missingness and temporal variation.

**Conclusion:**

An interpretable gradient-boosting model may support risk-stratified perioperative assessment for elderly patients undergoing abdominal surgery. Prospective multicenter validation is required before routine clinical implementation.

## 1 Introduction

Postoperative pulmonary complications (PPCs) are frequent adverse events after abdominal surgery and are associated with prolonged hospitalization, intensive care use, increased costs, and excess mortality (1, 2). Older adults are particularly vulnerable because age-related respiratory reserve, comorbidity burden, frailty-related functional limitation, and surgical stress may interact to impair postoperative ventilation and airway protection (1, 3). Reported PPC incidence varies across populations and definitions, but clinically important event rates around 15%–20% are common in elderly or major abdominal surgery cohorts (2–5).

Several perioperative risk tools, including ARISCAT and NSQIP-based calculators, are available for PPC risk estimation (2, 4). However, their performance can vary across institutions and case mixes, and some require variables that are not uniformly available in urgent surgical pathways. This issue is relevant for Chinese tertiary surgical settings, where local surgical volume, patient profiles, and perioperative workflows may differ from those represented in derivation cohorts.

Machine learning offers a way to model non-linear and interacting clinical predictors, but prediction accuracy alone is insufficient for perioperative decision support. Models intended for clinical use must be transparent, calibrated, externally assessed, and useful across plausible decision thresholds (6–13). SHAP-based explanations can help clinicians understand how individual features contribute to predicted risk while preserving the flexibility of gradient-boosting algorithms (14–16).

This study aimed to develop and validate machine learning models for 30-day PPCs in patients aged > = 65 years undergoing abdominal surgery. The planned primary outcome was the occurrence of any PPC within 30 postoperative days. Secondary and sensitivity objectives were to compare model performance with traditional regression and existing scores, assess external validation and clinical utility, describe PPC severity and co-occurrence patterns, evaluate missing pulmonary function data, and develop an interpretable prototype risk tool for preoperative or early perioperative use.

## 2 Materials and methods

### 2.1 Study design, setting, ethics, and sample-size rationale

This retrospective prediction-model study used routinely collected perioperative data from elderly patients who underwent abdominal surgery between January 2018 and December 2022. The development/internal cohort comprised patients treated at Ganzhou Hospital-Nanfang Hospital, Southern Medical University (Ganzhou People's Hospital). An independent external-validation cohort from a collaborating institution was used to assess model transportability.

The model was developed to predict 30-day postoperative pulmonary complications (PPCs). Before applying the eligibility criteria, the anticipated PPC event rate was assumed to be approximately 15%–20% based on previous perioperative studies in elderly or major abdominal surgery populations and on validation studies of PPC risk models (2–5). This assumption was consistent with the observed event rate in the development/internal cohort. The development/internal cohort included 2,456 patients with 425 PPC events, providing more than 15 events per candidate predictor parameter for the approximately 28 candidate predictor parameters considered during model development. This event count was considered adequate for model development, internal validation, and sensitivity analyses according to contemporary prediction-model sample-size guidance (6–9).

The study was approved by the Institutional Review Board of Ganzhou Hospital-Nanfang Hospital, Southern Medical University (approval no. gh2501223), and informed consent was waived because of the retrospective and de-identified nature of the study. All procedures conformed to the Declaration of Helsinki and applicable data-protection requirements.

### 2.2 Study population and eligibility criteria

Patients were eligible if they were aged ≥ 65 years, underwent elective or emergency abdominal surgery under general anesthesia, and had sufficient perioperative records for candidate predictors and postoperative outcome ascertainment. Abdominal surgery was defined as any intra-abdominal procedure involving gastrointestinal, hepatobiliary, pancreatic, urological, or gynecological operations.

Patients were excluded if they had previous lung resection or pneumonectomy, pre-existing tracheostomy or chronic mechanical ventilation, concurrent thoracic surgery, ASA physical status class VI, more than 30% missingness across key candidate predictors, or intraoperative or first-24-hour death from non-pulmonary causes. These exclusions were applied before model development and validation.

### 2.3 Candidate predictors

Candidate predictors were selected before model development based on clinical plausibility, previous PPC literature, and routine perioperative availability. They included demographic characteristics, smoking history, ASA physical status, functional dependence, respiratory and cardiovascular comorbidities, metabolic and renal comorbidities, Charlson Comorbidity Index, selected laboratory markers, pulmonary function parameters when available, surgical urgency, surgical site, surgical approach, operative duration, estimated blood loss, and transfusion. ASA physical status was treated as a global perioperative physical-status measure rather than a direct frailty measure.

### 2.4 PPC ascertainment and severity classification

PPCs were ascertained within 30 postoperative days using EPCO and related perioperative pulmonary endpoint definitions. Events included pneumonia, respiratory failure, pleural effusion requiring drainage, atelectasis requiring therapeutic intervention, pneumothorax, bronchospasm requiring treatment, ARDS according to the Berlin definition, aspiration pneumonitis, postoperative positive-pressure ventilation, unplanned reintubation, and prolonged mechanical ventilation >48 h (5, 17, 18).

Potential PPC events were reviewed from clinical notes, respiratory therapy records, radiology reports, laboratory results, and medication records. Disagreements were resolved by consensus. Major PPC was defined as respiratory failure, ARDS, unplanned reintubation, or prolonged mechanical ventilation >48 h; other PPCs without a major component were classified as minor PPC.

### 2.5 Missing data and sensitivity analyses

Missingness was summarized for all candidate predictors and comparator scores. Most routinely available clinical predictors used in the primary model were complete or nearly complete. Pulmonary function variables had moderate missingness and were therefore evaluated in sensitivity analyses. Multiple imputation by chained equations was performed with 10 imputed datasets for the pulmonary-function sensitivity analysis; the imputation model included the outcome, routinely available clinical predictors, pulmonary function variables, and auxiliary clinical variables, but the outcome itself was not imputed (10). ARISCAT and NSQIP respiratory risk scores were treated as comparator tools rather than candidate predictors and were evaluated in complete-score subsets instead of being imputed.

### 2.6 Model development, validation, and interpretation

The development/internal dataset was partitioned into training, internal-validation, and independent-test sets by stratified sampling. Six algorithms were compared: penalized logistic regression, random forest, XGBoost, LightGBM, support vector machine, and artificial neural network (15, 16, 19–22). Class imbalance was addressed only within the training set using SMOTE to avoid leakage (20). Hyperparameters were tuned by cross-validation and Bayesian optimization. Model performance was assessed using AUC, bootstrap 95% confidence intervals, sensitivity, specificity, positive predictive value, negative predictive value, Brier score, calibration plots, DeLong comparisons, and decision curve analysis (11–13). The external-validation cohort was held out from model training.

The best-performing model was interpreted using SHAP values to quantify global and patient-level feature contributions (14). A simplified nomogram and a prototype web-based calculator were developed to demonstrate potential clinical usability. These tools were designed for preoperative or early perioperative risk communication and were not presented as evidence of prospective clinical impact.

### 2.7 Statistical analysis

Continuous variables are presented as mean (SD) or median (IQR), and categorical variables as *n* (%). Group comparisons used t tests or Wilcoxon rank-sum tests for continuous variables and chi-square or Fisher exact tests for categorical variables, as appropriate. Standardized mean differences were used to compare development/internal and external-validation cohorts. Additional analyses examined PPC co-occurrence patterns, minor versus major PPC outcomes, model performance by year, and calendar-year/COVID-era sensitivity models. Two-sided *P* < 0.05 was considered statistically significant. Analyses were conducted in R and Python.

## 3 Results

### 3.1 Study population and postoperative outcomes

**Figure 1:**
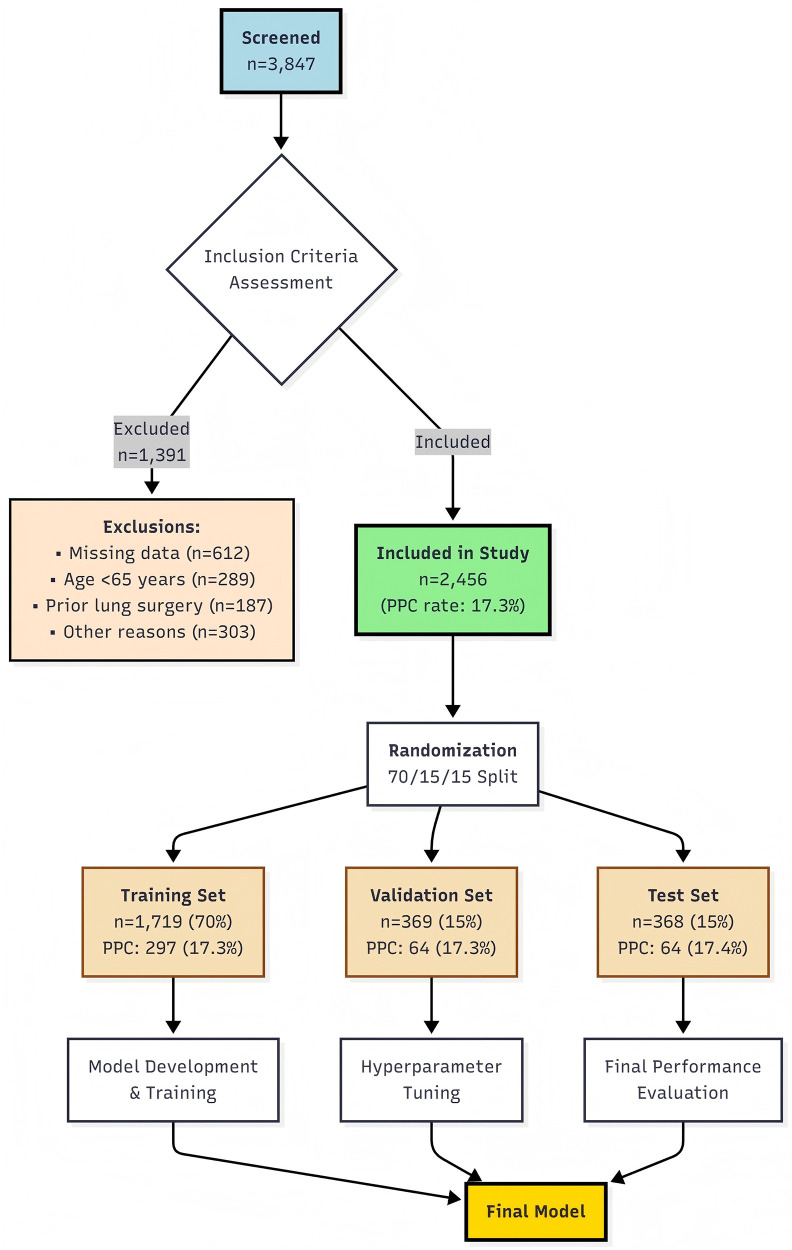
Patient selection flowchart. The diagram summarizes screening, exclusion, and cohort allocation for the development/internal and external-validation datasets. PPC, postoperative pulmonary complications.

Among 3,847 screened patients, 2,456 patients were included in the development/internal cohort and 542 in the external-validation cohort (Figure 1). PPCs occurred in 425 development/internal patients (17.3%) and 105 external-validation patients (19.4%). Baseline differences between patients with and without PPCs were clinically plausible and included older age, higher COPD prevalence, more emergency surgery, more upper abdominal surgery, lower albumin, and longer operative duration (Table 1). Patients with PPCs had longer hospital stay, more ICU admission, more mechanical ventilation, higher 30- and 90-day mortality, more readmissions, and higher hospital costs (Table 2). External-validation baseline characteristics are provided in Supplementary Table S1.

**Table 1:** Baseline characteristics stratified by postoperative pulmonary complications.

Characteristic	No PPC (*n* = 2,031)	PPC (*n* = 425)	*P*-value
Age, years, mean (SD)	73.4 (6.1)	76.8 (7.4)	<0.001
Age > = 85 years, *n* (%)	139 (6.8)	72 (16.9)	<0.001
Male sex, *n* (%)	959 (47.2)	249 (58.6)	<0.001
BMI, kg/m2, mean (SD)	26.5 (4.7)	25.4 (5.1)	0.002
Current smoker, *n* (%)	261 (12.9)	103 (24.2)	<0.001
COPD, *n* (%)	250 (12.3)	134 (31.5)	<0.001
Heart failure, *n* (%)	191 (9.4)	97 (22.8)	<0.001
Diabetes mellitus, *n* (%)	578 (28.5)	153 (36.0)	0.002
Chronic kidney disease, *n* (%)	317 (15.6)	122 (28.7)	<0.001
ASA class III-IV, *n* (%)	787 (38.7)	212 (49.9)	<0.001
Functional dependence, *n* (%)	276 (13.6)	111 (26.1)	<0.001
Albumin, g/L, mean (SD)	38.6 (4.1)	35.2 (4.8)	<0.001
FEV1, % predicted, mean (SD)	84.1 (16.7)	69.8 (20.1)	<0.001
Emergency surgery, *n* (%)	246 (12.1)	121 (28.5)	<0.001
Upper abdominal site, *n* (%)	790 (38.9)	264 (62.1)	<0.001
Open approach, *n* (%)	856 (42.1)	268 (63.1)	<0.001
Duration, min, median (IQR)	169 (120–241)	246 (174–348)	<0.001

Values are mean (SD), median (IQR), or *n* (%). *P* values compare patients with and without PPC.

PPC, postoperative pulmonary complications; BMI, body mass index; COPD, chronic obstructive pulmonary disease; ASA, American Society of Anesthesiologists; FEV1, forced expiratory volume in 1 s.

**Table 2:** Clinical outcomes stratified by postoperative pulmonary complications.

Outcome	No PPC (*n* = 2,031)	PPC (*n* = 425)	*P*-value
Hospital LOS, days, median (IQR)	7 (5–10)	18 (14–25)	<0.001
ICU admission, *n* (%)	256 (12.6)	201 (47.3)	<0.001
Mechanical ventilation, *n* (%)	87 (4.3)	156 (36.7)	<0.001
30-day mortality, *n* (%)	56 (2.8)	54 (12.7)	<0.001
90-day mortality, *n* (%)	68 (3.3)	78 (18.4)	<0.001
Hospital readmission, *n* (%)	187 (9.2)	98 (23.1)	<0.001
Total costs, CNY, median (IQR)	23,490 (14,965–38,985)	69,700 (47,460–100,780)	<0.001

Values are median (IQR) or *n* (%).

LOS, length of stay; ICU, intensive care unit; CNY, Chinese yuan.

Pneumonia, respiratory failure, and atelectasis were the most common PPC components. Of 425 patients with PPCs, 121 (28.5%) had more than one pulmonary complication. The most common co-occurrence patterns are shown in Supplementary Table S6 and Supplementary Figure S4. Analyses stratified by minor and major PPC are presented in Supplementary Table S7.

### 3.2 Model performance and validation

In the independent test set, XGBoost had the highest overall performance among the models evaluated, with an AUC of 0.856 (95% CI, 0.811–0.900) and a Brier score of 0.107 (Table 3). At the prespecified 20% risk threshold, sensitivity, specificity, PPV, and NPV were 87.5%, 60.9%, 32.0%, and 95.9%, respectively. LightGBM showed similar but slightly lower discrimination, whereas logistic regression had lower AUC and Brier performance.

**Table 3:** Independent-test performance of the three main comparator models.

Model	AUC (95% CI)	Sensitivity (%)	Specificity (%)	PPV (%)	NPV (%)	Brier score
XGBoost	0.856 (0.811–0.900)	87.5	60.9	32.0	95.9	0.107
LightGBM	0.836 (0.782–0.883)	85.9	60.5	31.4	95.3	0.110
Logistic regression	0.712 (0.641–0.783)	67.2	68.4	30.9	90.8	0.131

AUC and 95% confidence intervals were estimated in the independent test set. Threshold-based metrics were calculated at the 20% predicted-risk threshold.

PPV, positive predictive value; NPV, negative predictive value.

**Figure 2:**
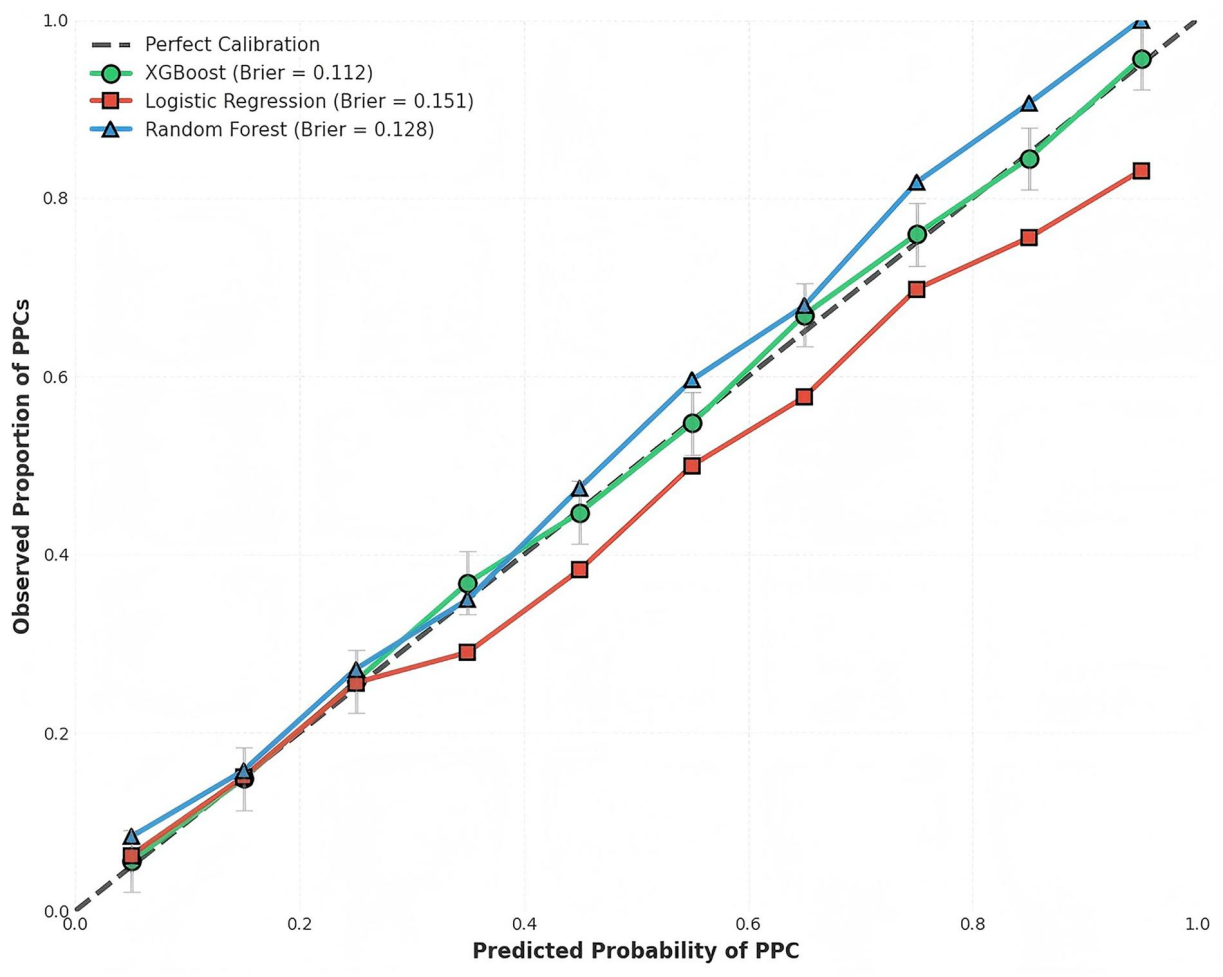
Calibration of the XGBoost model. Calibration plots compare observed PPC risk with predicted risk across risk deciles in the independent test and external-validation cohorts. The diagonal line represents perfect calibration.

**Figure 3:**
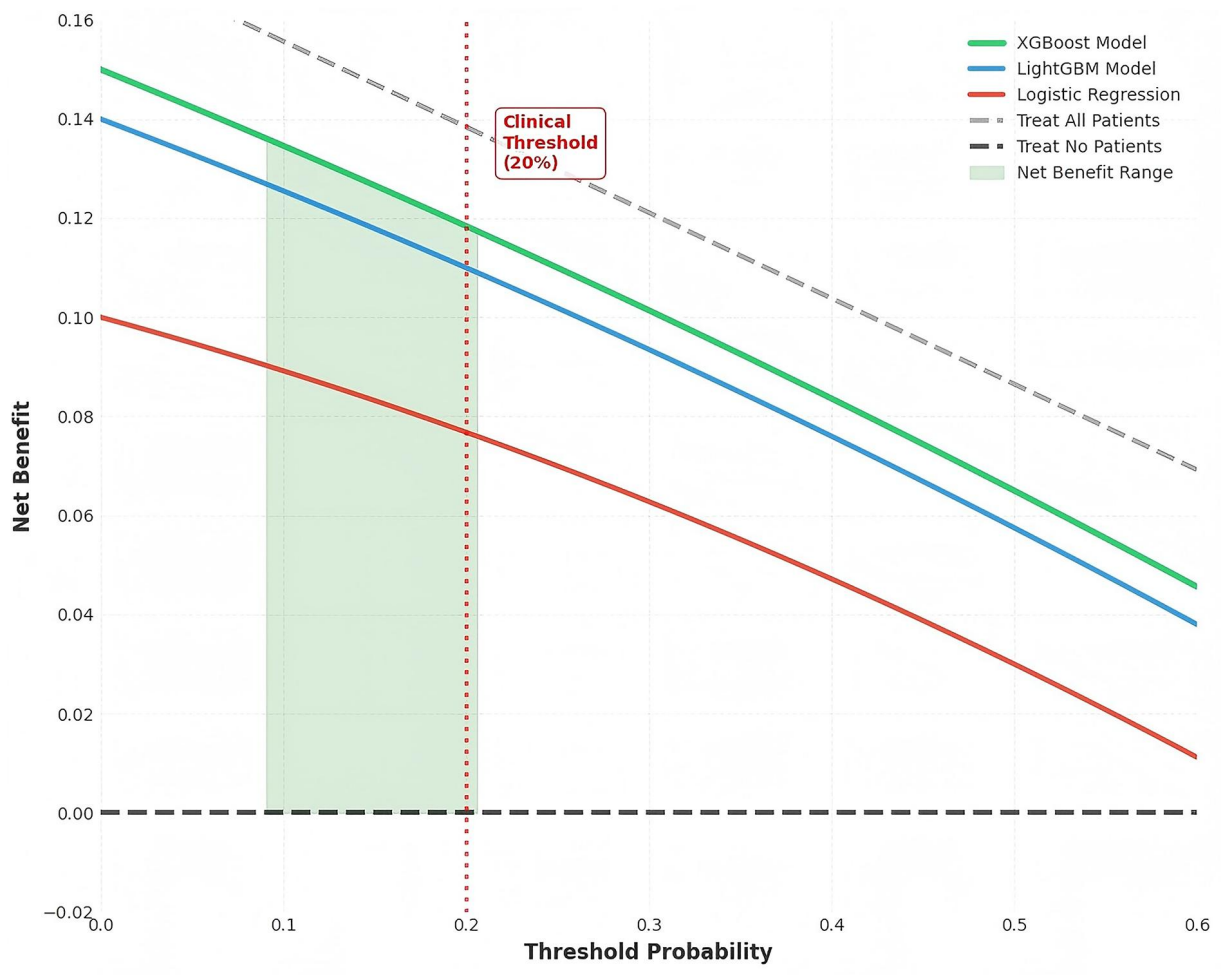
Decision curve analysis of model-predicted PPC risk. Net benefit is shown across clinically plausible threshold probabilities, comparing XGBoost with logistic regression and default treat-all/treat-none strategies.

Across training, internal-validation, independent-test, and external-validation datasets, XGBoost AUCs were 0.833, 0.820, 0.856, and 0.821, respectively (Table 4). This pattern did not suggest major overfitting. Calibration was acceptable overall (Figure 2), although decile-level fluctuations were observed because of limited event counts within some risk strata (Supplementary Figure S2). Decision curve analysis (Figure 3) showed higher net benefit for XGBoost than logistic regression and treat-all/treat-none strategies across clinically relevant thresholds, particularly around 10%–30%.

**Table 4:** XGBoost performance across development, test, and external-validation datasets.

Validation set	*n*	PPC, *n* (%)	AUC (95% CI)	Brier score
Training	1,719	297 (17.3)	0.833 (0.812–0.854)	0.113
Internal validation	369	64 (17.3)	0.820 (0.774–0.866)	0.117
Independent test	368	64 (17.4)	0.856 (0.811–0.900)	0.107
External validation	542	105 (19.4)	0.821 (0.781–0.861)	0.122

The external-validation cohort was not used for model training or hyperparameter tuning.

### 3.3 Model interpretation and risk stratification

**Figure 4:**
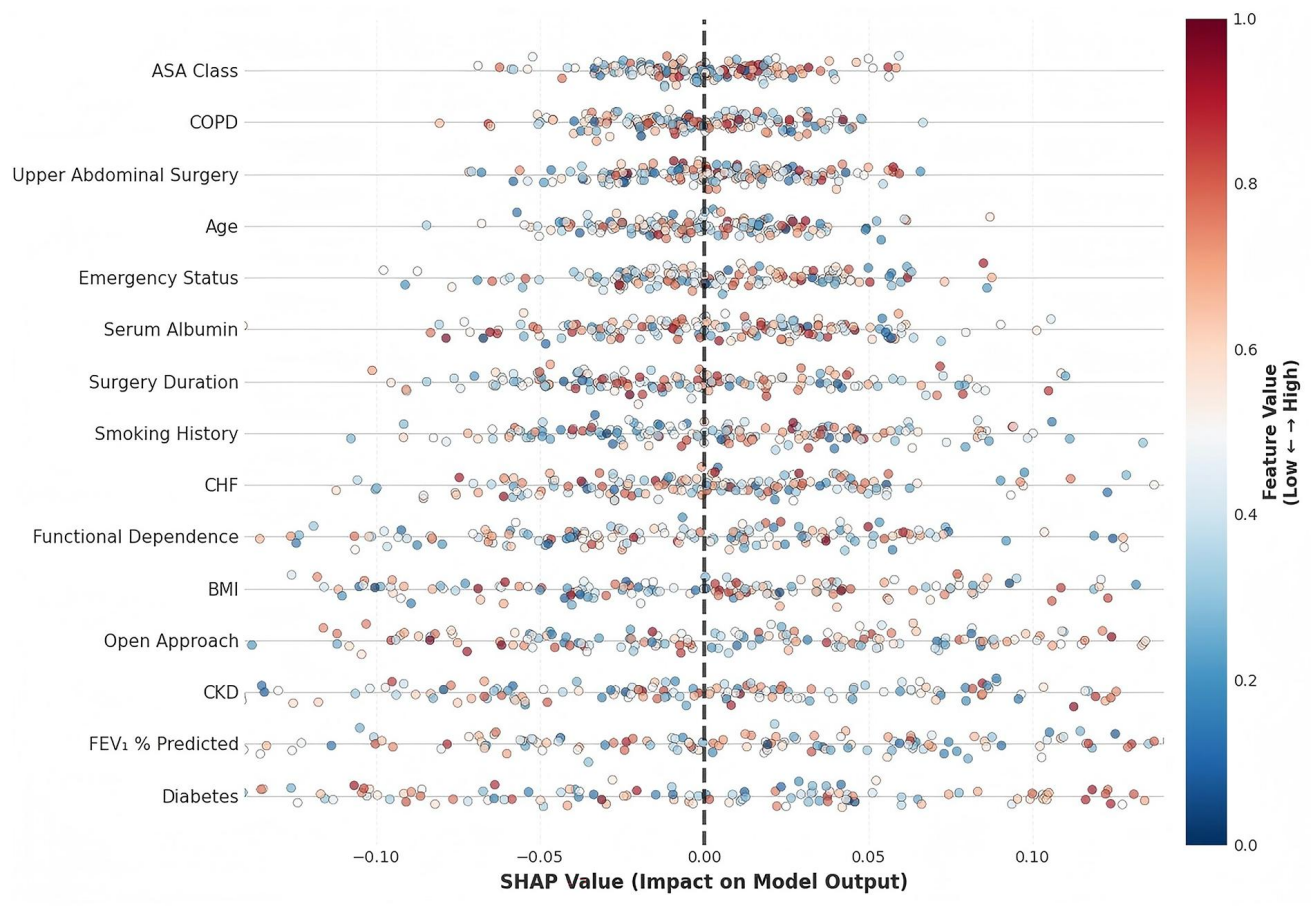
SHAP summary plot for the XGBoost model. Features are ordered by mean absolute SHAP value. Each point represents one patient; color intensity indicates feature value, and horizontal position indicates the direction and magnitude of contribution to predicted PPC risk.

**Figure 5:**
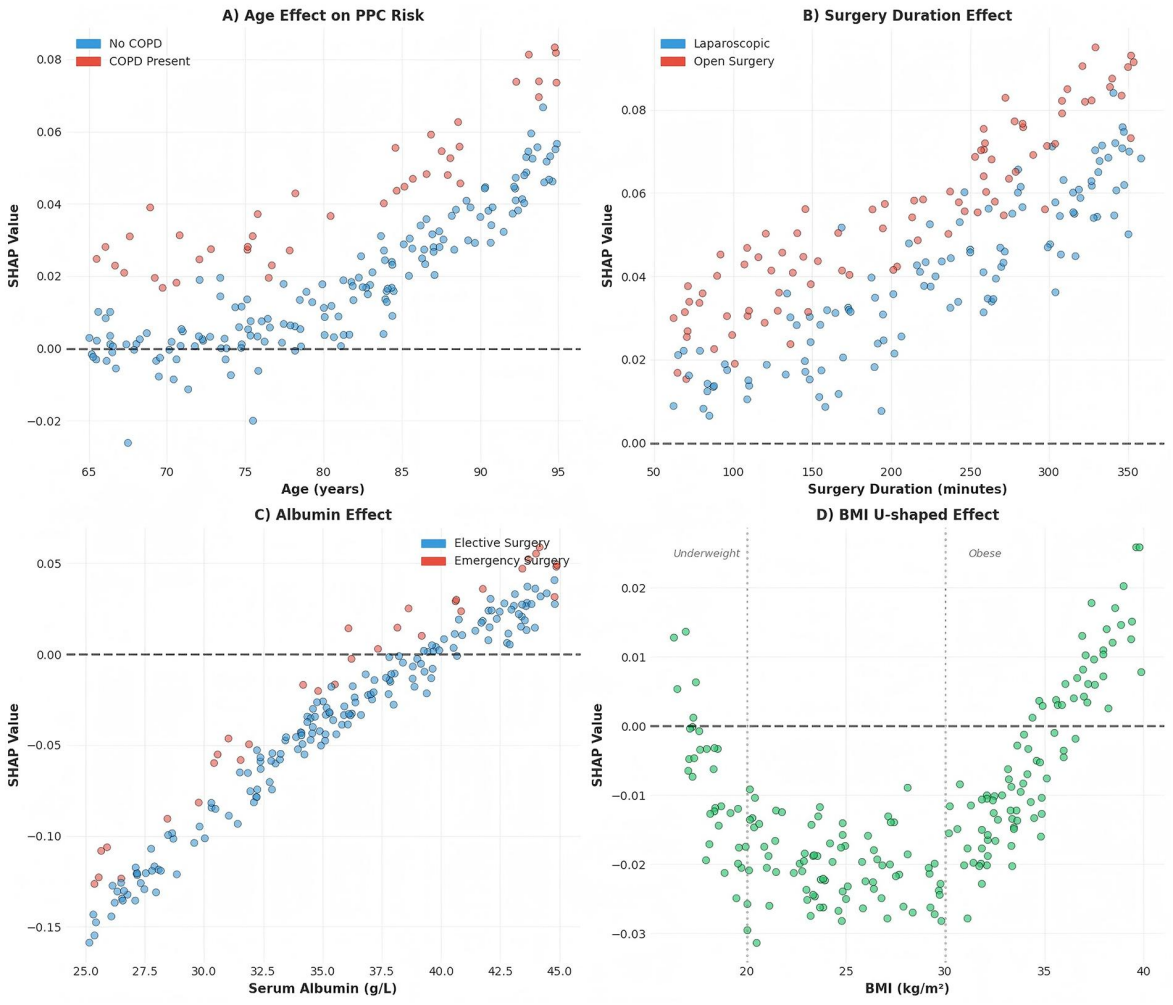
SHAP dependence plots for selected predictors. **(A)** Age effect on PPC risk stratified by COPD status. **(B)** Surgery duration effect stratified by surgical approach. **(C)** Albumin effect stratified by elective versus emergency surgery. **(D)** BMI effect showing a non-linear U-shaped association. The plots illustrate non-linear relationships between selected predictors and predicted PPC risk while accounting for interactions learned by the XGBoost model.

SHAP analysis identified ASA physical status, COPD, upper abdominal site, age, emergency surgery, albumin, operative duration, smoking, heart failure, and functional dependence as leading contributors to predicted PPC risk (Figures 4, 5). Emergency surgery was retained as a risk marker rather than a directly modifiable target. The overlap between XGBoost predictors and known clinical risk factors supports face validity; the added value of the model was its ability to combine these predictors non-linearly and provide individualized explanations.

**Figure 6:**
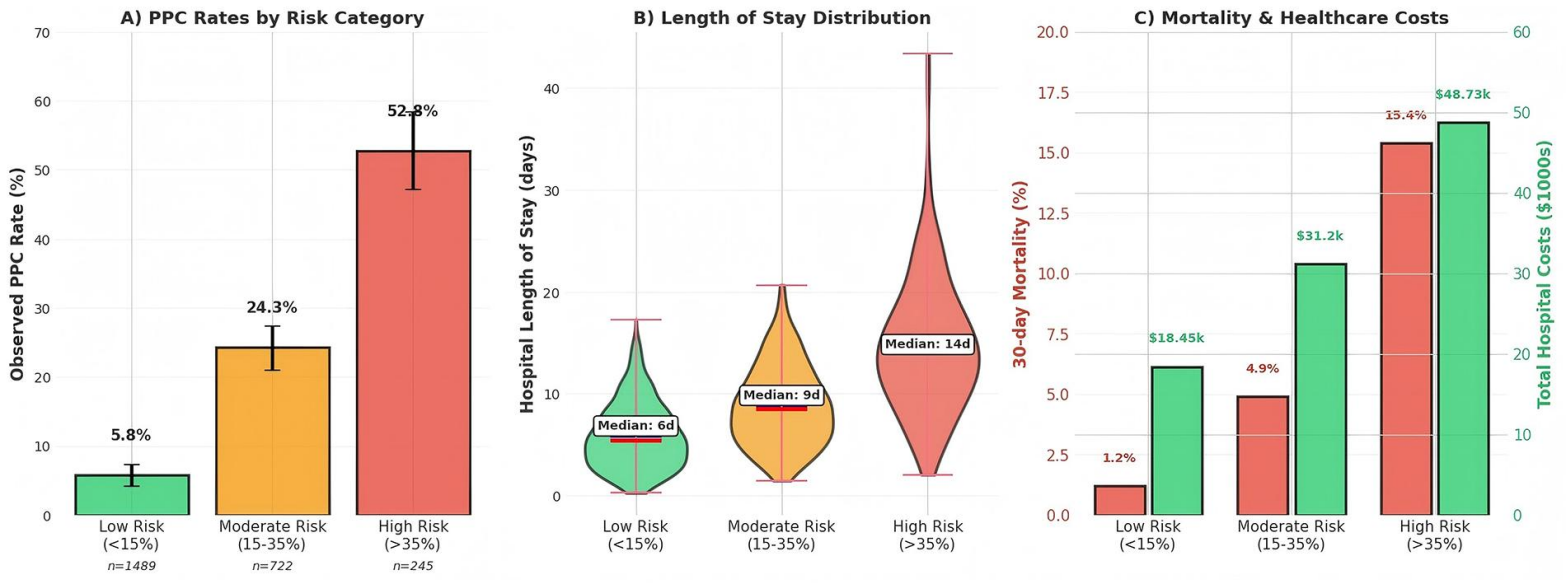
Observed outcomes across XGBoost-defined risk strata. **(A)** Observed PPC rates by predicted-risk category. **(B)** Hospital length-of-stay distributions by predicted-risk category. **(C)** Thirty-day mortality and total hospital costs by predicted-risk category. Patients were grouped into low-, moderate-, and high-risk categories according to predicted PPC probability.

XGBoost stratified patients into low-, moderate-, and high-risk groups with observed PPC rates of 6.9%, 24.4%, and 37.6%, respectively (Table 5; Figure 6). This gradient also corresponded to progressively higher ICU admission, mortality, and hospital costs. Comparator-score analyses are shown in Table 6 and Supplementary Table S8. In external validation, XGBoost showed numerically higher discrimination than logistic regression and available comparator scores; comparator-score analyses were restricted to patients with complete score components and should be interpreted as exploratory.

**Table 5:** Patient distribution and outcomes across XGBoost risk strata.

Outcome	Low risk (*n* = 1,254)	Moderate risk (*n* = 856)	High risk (*n* = 346)	*P*-value
PPC, *n* (%)	86 (6.9)	209 (24.4)	130 (37.6)	<0.001
LOS, days, median (IQR)	6 (5–9)	11 (7–17)	12 (6–20)	<0.001
ICU admission, *n* (%)	103 (8.2)	209 (24.4)	145 (41.9)	<0.001
30-day mortality, *n* (%)	15 (1.2)	42 (4.9)	53 (15.3)	<0.001
Total costs, CNY, median	18,830	37,985	62,010	<0.001

Risk strata were defined by predicted PPC probability: low risk <15%, moderate risk 15%–35%, and high risk >35%.

LOS, length of stay; ICU, intensive care unit.

**Table 6:** Discrimination of XGBoost, logistic regression, and available comparator scores.

Cohort/model	*n*	PPC events	AUC (95% CI)	Comment
Development/internal XGBoost	2,456	425	0.833 (0.816–0.851)	Primary model, full cohort
Development/internal logistic regression	2,456	425	0.693 (0.665–0.721)	Comparator model
Development/internal ARISCAT subset	1,247	218	0.736 (0.700–0.773)	Complete-score subset
Development/internal NSQIP subset	1,832	313	0.679 (0.644–0.714)	Complete-score subset
External-validation XGBoost	542	105	0.821 (0.781–0.861)	Independent external cohort
External-validation logistic regression	542	105	0.699 (0.642–0.755)	Comparator model
External-validation ARISCAT subset	282	52	0.707 (0.627–0.788)	Exploratory complete-score subset
External-validation NSQIP subset	410	76	0.724 (0.660–0.787)	Exploratory complete-score subset

ARISCAT and NSQIP analyses were restricted to patients with complete components for each score. External ARISCAT and NSQIP comparisons were considered exploratory because of smaller complete-score subsets.

### 3.4 Missing data, temporal stability, and other sensitivity analyses

Pulmonary function variables had 18.7% missingness. In the MICE sensitivity analysis, adding imputed FEV1% predicted and FEV1/FVC ratio to the core clinical model increased apparent AUC from 0.8426 to 0.8769 and reduced the Brier score from 0.1022 to 0.0896 across 10 imputed datasets. A complete-case PFT analysis showed a similar direction (AUC 0.8400 for the core model versus 0.8732 for the core plus PFT model). These findings support the relevance of PFT information but also justify the primary model emphasis on routinely available predictors when PFTs are unavailable in urgent or resource-limited pathways (Supplementary Tables S2-S5).

Annual PPC rates decreased numerically from 19.0% in 2018 to 15.8% in 2022, while annual XGBoost AUCs remained generally stable (0.797–0.865). Calendar-year trend and COVID-era sensitivity models did not show statistically significant associations after adjustment (Supplementary Tables S9 and S10; Supplementary Figure S5). These analyses support temporal robustness but do not eliminate the possibility of unmeasured practice changes during the COVID-19 period.

### 3.5 Prototype clinical tool

**Figure 7:**
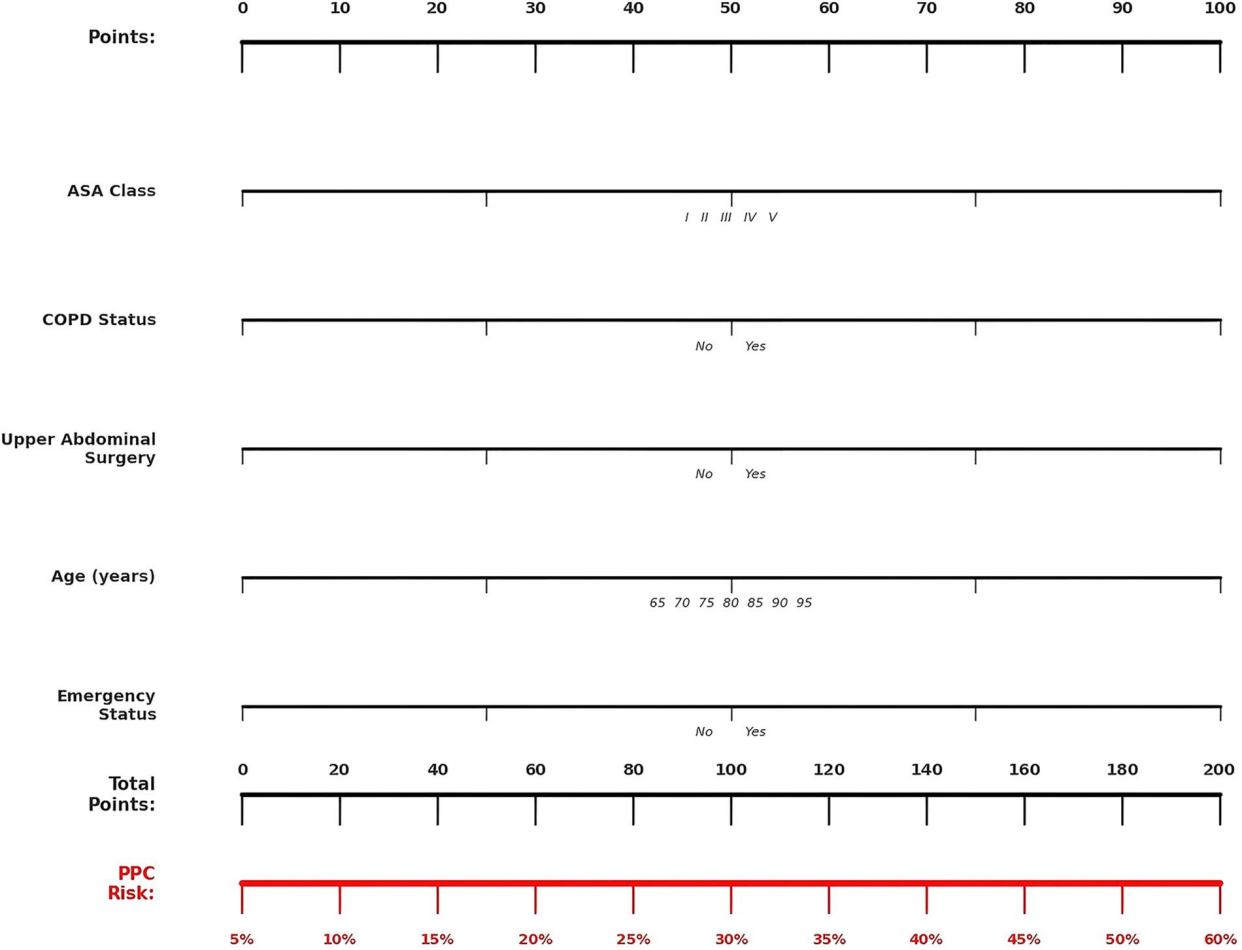
Simplified nomogram for bedside PPC risk estimation. The nomogram was derived from leading model predictors and is intended as a practical illustrative tool rather than evidence of prospective implementation.

**Figure 8:**
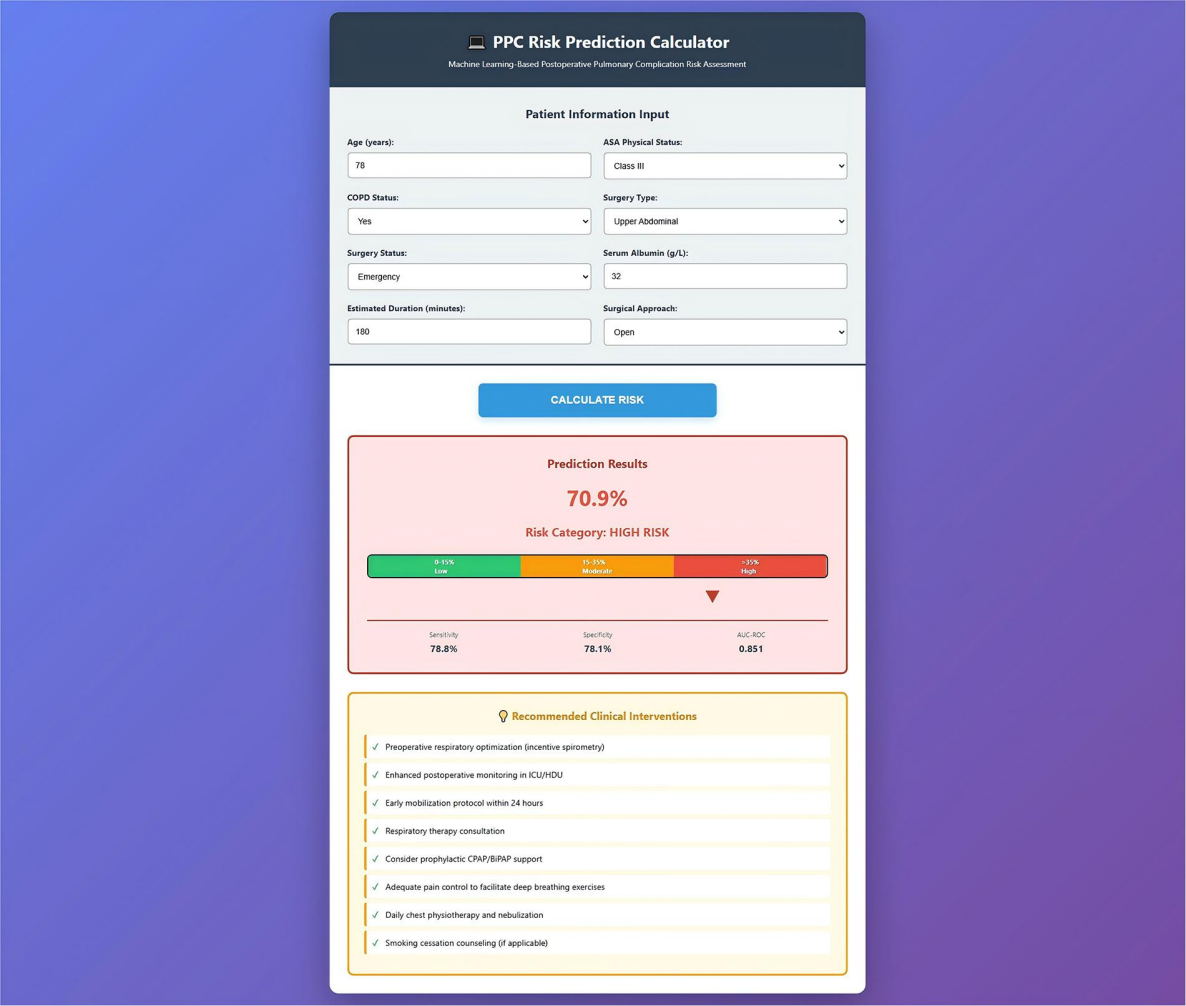
Prototype web-based risk calculator interface. The prototype displays predicted PPC probability and patient-level feature contributions for preoperative or early perioperative planning.

**Figure 9:**
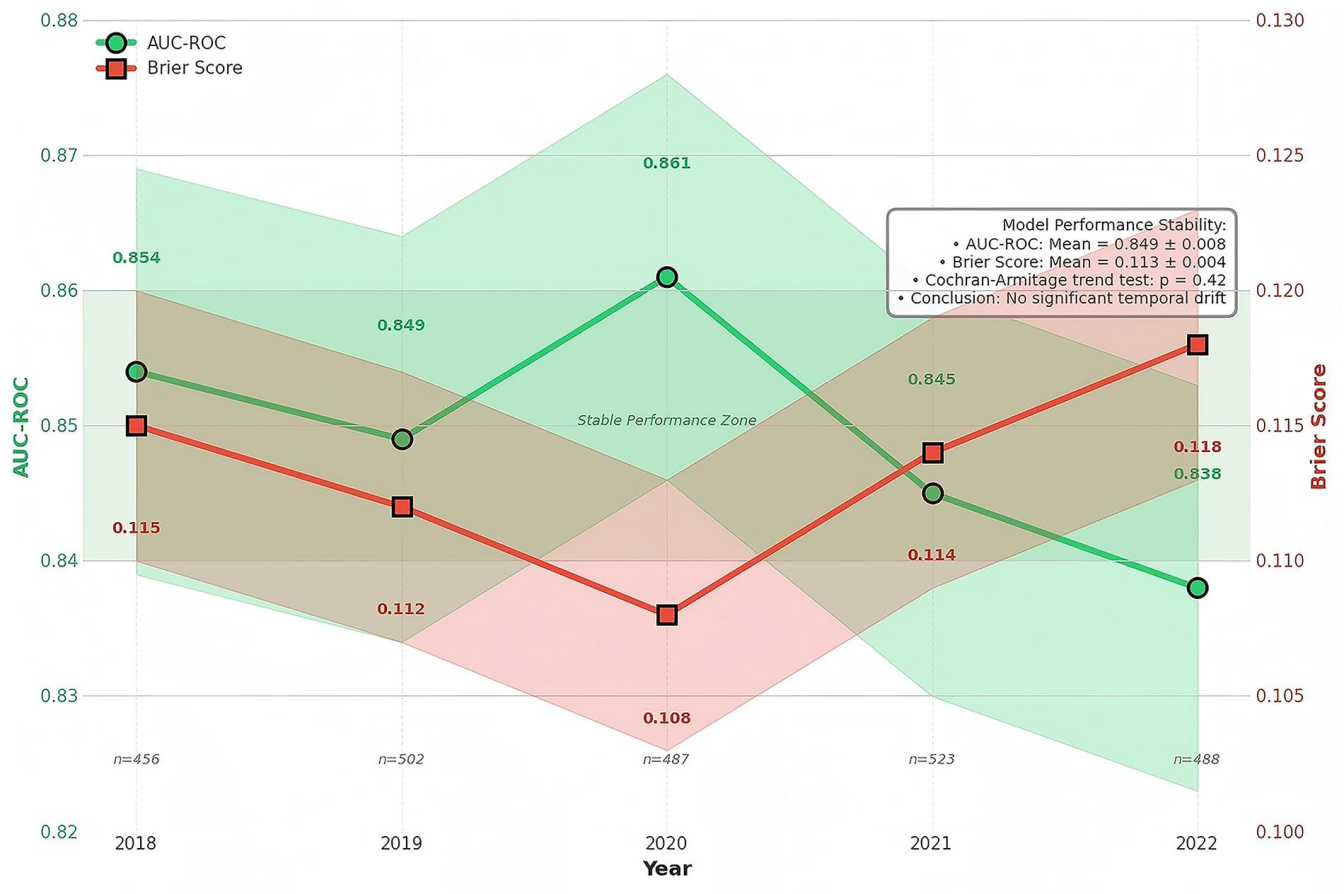
Temporal pattern of PPC incidence and model performance. Annual PPC incidence and discrimination were examined across the 2018−2022 study period to assess temporal stability and contextual effects.

A simplified nomogram based on the leading predictors was generated to illustrate bedside risk estimation, and a prototype web calculator was developed to demonstrate how patient-level risk contributions could be displayed (Figures 7, 8). Temporal model performance across study years is summarized in Figure 9. These materials are intended as implementation prototypes rather than evidence of prospective clinical effectiveness. The appropriate time of use is during preoperative assessment or early perioperative planning, after surgical urgency, site, approach, ASA class, and key laboratory values are known.

## 4 Discussion

### 4.1 Principal findings

The present study developed and externally assessed an interpretable machine-learning model for predicting 30-day postoperative pulmonary complications (PPCs) in elderly patients undergoing abdominal surgery. XGBoost showed better discrimination and clinical utility than logistic regression and numerically better performance than available comparator scores, while maintaining acceptable performance in the external-validation cohort. The model also separated patients into clinically meaningful risk strata and provided SHAP-based explanations of individual predictions. These findings are relevant because perioperative prediction models should be evaluated not only by discrimination, but also by calibration, validation, clinical utility, transparency, and intended clinical use.

The strongest predictors identified by the model—ASA physical status, COPD, upper abdominal surgery, age, emergency surgery, albumin, and operative duration—are established perioperative risk markers rather than entirely novel findings (1–4, 23). Therefore, the main contribution of this work is not the discovery of new risk factors, but the integration of familiar and routinely available predictors into a non-linear, explainable model. This distinction is important: in clinical prediction, practical value often comes from improved individual risk estimation and workflow feasibility rather than from identifying previously unknown predictors.

The observed clinical burden of PPCs was consistent with prior perioperative literature. PPCs are heterogeneous events ranging from atelectasis and pneumonia to respiratory failure, prolonged ventilation, and acute respiratory distress syndrome (1, 5, 17, 18). Previous multicenter evidence has shown that PPCs, including less severe respiratory events, are associated with increased early mortality, ICU admission, and hospital length of stay (23). In the present cohort, PPCs were similarly associated with longer hospitalization, greater ICU use, higher mortality, and higher hospital costs. The additional minor/major PPC and co-occurrence analyses provided further context for interpreting the composite endpoint.

### 4.2 Clinical and methodological implications

Risk stratification may help clinicians prioritize respiratory optimization, postoperative monitoring, physiotherapy, and shared decision-making in elderly surgical patients. However, the model should be viewed as decision support rather than decision replacement. Some predictors, such as age, emergency surgery, and ASA physical status, are not directly modifiable, but they can identify patients who may need intensified surveillance. Other factors, including respiratory status, nutritional status, surgical approach, and perioperative ventilation strategy, may be more actionable in selected patients. Evidence from perioperative trials supports the potential role of lung-protective ventilation and preoperative physiotherapy in reducing respiratory complications in appropriate abdominal surgery populations (24, 25). The present study, however, did not test a model-guided intervention pathway.

Decision curve analysis suggested that XGBoost provided higher net benefit than logistic regression and default treat-all or treat-none strategies across clinically relevant threshold ranges. This supports the potential usefulness of the model for selecting patients who may benefit from additional respiratory assessment or monitoring (12, 13). Still, DCA does not prove that model-guided care improves outcomes. Prospective implementation studies are needed to determine whether using the model changes management, reduces PPCs, or improves resource allocation.

The missing-data sensitivity analyses clarified the role of pulmonary function testing. PFT variables improved apparent performance in sensitivity analyses, which is clinically plausible because reduced FEV₁, FVC, and FEV₁/FVC ratio reflect impaired respiratory reserve. However, PFTs were not universally available, particularly in emergency or resource-limited settings. The primary model therefore prioritized routinely available clinical variables, while PFT-enhanced models were presented as supportive sensitivity analyses. This approach improves practicality while acknowledging the additional prognostic signal of objective pulmonary function when available (9, 10).

SHAP analysis improved interpretability by showing both global feature importance and individual-level predictor contributions (14). The SHAP findings were clinically coherent, with established perioperative risk markers driving most predictions. This increases face validity and may improve clinician trust. Nevertheless, SHAP explains model behavior rather than proving causal relationships. Similarly, although gradient-boosting algorithms can capture non-linear associations and interactions, they may overfit if validation, calibration, and missing-data handling are inadequate (8, 9, 26). For this reason, the model was assessed using external validation, calibration analysis, decision-curve analysis, temporal/COVID-era sensitivity analyses, PFT missing-data analyses, and PPC severity analyses.

### 4.3 Limitations

This study has several limitations. First, the development dataset was retrospective and derived from a single tertiary center. Although the external-validation cohort improves rigor, prospective multicenter validation remains necessary before routine clinical use. Second, the PPC endpoint was a composite outcome including events of different severities. Minor/major PPC analyses and co-occurrence summaries were therefore provided, but residual heterogeneity remains (5, 17). Third, missingness in pulmonary function variables and comparator-score components followed clinical patterns rather than purely random mechanisms. MICE and complete-case sensitivity analyses partly addressed this issue but cannot eliminate all missing-data bias. Fourth, calendar-year and COVID-era analyses were exploratory and could not fully capture changes in surgical selection, perioperative workflow, infection-control practice, or postoperative monitoring during 2018–2022. Fifth, the nomogram and web calculator should be considered prototypes. We did not conduct a prospective clinician-use study, randomized implementation trial, or formal health-economic evaluation. Therefore, the cost and length-of-stay findings should be interpreted as descriptive burden analyses rather than evidence of cost-effectiveness. Finally, although the model showed promising external performance, local recalibration may be needed before adoption in different hospitals or health systems (9, 27).

## 5 Conclusion

An interpretable XGBoost model showed promising performance for predicting 30-day PPCs in elderly patients undergoing abdominal surgery, with acceptable external validation, clinically meaningful risk stratification, and SHAP-based explanations. The model integrates established perioperative risk markers into a practical decision-support framework and may help identify patients who require closer respiratory assessment, optimization, and postoperative surveillance. Prospective multicenter validation and implementation studies are required before routine clinical adoption.

## Data Availability

The original contributions presented in the study are included in the article/Supplementary Material, further inquiries can be directed to the corresponding author.
