# Supplementary material for: Machine learning-based prediction models for postoperative pulmonary complications in elderly patients undergoing abdominal surgery
Source: Front Surg. 2026 Jul 14;13:1780650. doi: 10.3389/fsurg.2026.1780650 (PMC13410041; doi:10.3389/fsurg.2026.1780650)
Supplement: Supplementary file 1 [file Supplementaryfile1.docx]

# Supplementary material

**Supplementary Table S1. Baseline characteristics of the development/internal and external-validation cohorts.**

Values are mean (SD), median (Q1, Q3), or n (%). SMD, standardized mean difference.

| **Characteristic** | **Development/internal (N=2,456)** | **External validation (N=542)** | **SMD** |
| --- | --- | --- | --- |
| Age, years | 74.0 (6.5) | 75.4 (6.7) | 0.211 |
| Age >=85 years | 211 (8.6%) | 54 (10.0%) | 0.047 |
| Male sex | 1,208 (49.2%) | 277 (51.1%) | 0.038 |
| BMI, kg/m2 | 26.3 (4.8) | 26.2 (4.9) | 0.025 |
| Current smoker | 364 (14.8%) | 83 (15.3%) | 0.014 |
| Former smoker | 434 (17.7%) | 96 (17.7%) | 0.001 |
| COPD | 384 (15.6%) | 91 (16.8%) | 0.031 |
| Asthma | 123 (5.0%) | 30 (5.5%) | 0.024 |
| Obstructive sleep apnea | 143 (5.8%) | 33 (6.1%) | 0.011 |
| Heart failure | 288 (11.7%) | 68 (12.5%) | 0.025 |
| Coronary artery disease | 489 (19.9%) | 115 (21.2%) | 0.032 |
| Hypertension | 1,496 (60.9%) | 337 (62.2%) | 0.026 |
| Diabetes mellitus | 731 (29.8%) | 166 (30.6%) | 0.019 |
| Chronic kidney disease | 439 (17.9%) | 100 (18.5%) | 0.015 |
| Charlson comorbidity index | 1.8 (1.5) | 2.0 (1.5) | 0.115 |
| ASA class III-IV | 999 (40.7%) | 230 (42.4%) | 0.036 |
| Functional dependence | 387 (15.8%) | 89 (16.4%) | 0.018 |
| Albumin, g/L | 38.0 (4.4) | 37.7 (4.5) | 0.060 |
| FEV1, % predicted | 81.7 (18.1) | 81.4 (18.3) | 0.017 |
| FVC, % predicted | 87.8 (20.1) | 88.0 (20.8) | 0.010 |
| FEV1/FVC ratio | 0.9 (0.1) | 0.9 (0.1) | 0.031 |
| Emergency surgery | 367 (14.9%) | 87 (16.1%) | 0.031 |
| Upper abdominal site | 1,054 (42.9%) | 241 (44.5%) | 0.031 |
| Open approach | 1,124 (45.8%) | 255 (47.0%) | 0.026 |
| Duration, min | 181.0 (128.0, 262.0) | 181.0 (124.0, 260.0) | 0.001 |
| PPC within 30 days | 425 (17.3%) | 105 (19.4%) | 0.053 |

**Supplementary Table S2. Missingness of candidate predictors and comparator scores by cohort.**

Only variables with missingness and representative complete predictors are shown. ARISCAT and NSQIP were treated as comparator scores and were not imputed.

| **Cohort** | **Variable** | **N** | **Missing, n** | **Missing, %** |
| --- | --- | --- | --- | --- |
| Development/internal | ariscat_score | 2,456 | 1,209 | 49.2 |
| External validation | ariscat_score | 542 | 260 | 48.0 |
| Development/internal | nsqip_respiratory_risk_pct | 2,456 | 624 | 25.4 |
| External validation | nsqip_respiratory_risk_pct | 542 | 132 | 24.4 |
| Development/internal | fev1_fvc_ratio | 2,456 | 459 | 18.7 |
| Development/internal | fev1_percent_predicted | 2,456 | 459 | 18.7 |
| Development/internal | fvc_percent_predicted | 2,456 | 459 | 18.7 |
| External validation | fev1_fvc_ratio | 542 | 101 | 18.6 |
| External validation | fev1_percent_predicted | 542 | 101 | 18.6 |
| External validation | fvc_percent_predicted | 542 | 101 | 18.6 |
| Development/internal | age | 2,456 | 0 | 0.0 |
| External validation | age | 542 | 0 | 0.0 |
| Development/internal | albumin_g_l | 2,456 | 0 | 0.0 |
| External validation | albumin_g_l | 542 | 0 | 0.0 |
| Development/internal | asa_class | 2,456 | 0 | 0.0 |
| External validation | asa_class | 542 | 0 | 0.0 |
| Development/internal | asthma | 2,456 | 0 | 0.0 |
| External validation | asthma | 542 | 0 | 0.0 |
| Development/internal | blood_transfusion | 2,456 | 0 | 0.0 |
| External validation | blood_transfusion | 542 | 0 | 0.0 |
| Development/internal | bmi | 2,456 | 0 | 0.0 |
| External validation | bmi | 542 | 0 | 0.0 |
| Development/internal | charlson_comorbidity_index | 2,456 | 0 | 0.0 |
| External validation | charlson_comorbidity_index | 542 | 0 | 0.0 |
| Development/internal | chronic_kidney_disease | 2,456 | 0 | 0.0 |
| External validation | chronic_kidney_disease | 542 | 0 | 0.0 |
| Development/internal | copd | 2,456 | 0 | 0.0 |
| External validation | copd | 542 | 0 | 0.0 |
| Development/internal | coronary_artery_disease | 2,456 | 0 | 0.0 |
| External validation | coronary_artery_disease | 542 | 0 | 0.0 |

**Supplementary Table S3. MICE-based pulmonary function sensitivity analysis (m=10).**

The outcome was not imputed. The PFT model added imputed FEV1 % predicted and FEV1/FVC ratio to the core clinical model.

| **Model** | **Variable** | **OR** | **95% CI lower** | **95% CI upper** | **P value** | **FMI** | **Lambda** |
| --- | --- | --- | --- | --- | --- | --- | --- |
| Core clinical model | Age | 1.062 | 1.043 | 1.081 | <0.001 | 0.001 | 0.000 |
| Core clinical model | Male sex | 1.132 | 0.875 | 1.464 | 0.344 | 0.001 | 0.000 |
| Core clinical model | BMI | 0.960 | 0.935 | 0.985 | 0.002 | 0.001 | 0.000 |
| Core clinical model | Current smoker | 1.626 | 1.190 | 2.221 | 0.002 | 0.001 | 0.000 |
| Core clinical model | COPD | 2.543 | 1.895 | 3.412 | <0.001 | 0.001 | 0.000 |
| Core clinical model | Heart failure | 2.342 | 1.692 | 3.242 | <0.001 | 0.001 | 0.000 |
| Core clinical model | Diabetes mellitus | 0.936 | 0.713 | 1.228 | 0.632 | 0.001 | 0.000 |
| Core clinical model | Chronic kidney disease | 1.506 | 1.122 | 2.023 | 0.006 | 0.001 | 0.000 |
| Core clinical model | asa_class2 | 0.918 | 0.571 | 1.476 | 0.724 | 0.001 | 0.000 |
| Core clinical model | ASA class III | 0.826 | 0.502 | 1.359 | 0.452 | 0.001 | 0.000 |
| Core clinical model | ASA class IV | 1.117 | 0.647 | 1.928 | 0.692 | 0.001 | 0.000 |
| Core clinical model | Functional dependence | 1.465 | 1.077 | 1.993 | 0.015 | 0.001 | 0.000 |
| Core clinical model | Albumin, g/L | 0.834 | 0.809 | 0.859 | <0.001 | 0.001 | 0.000 |
| Core clinical model | Emergency surgery | 1.800 | 1.332 | 2.434 | <0.001 | 0.001 | 0.000 |
| Core clinical model | Upper abdominal site | 1.744 | 1.352 | 2.250 | <0.001 | 0.001 | 0.000 |
| Core clinical model | Open approach | 1.527 | 1.178 | 1.980 | 0.001 | 0.001 | 0.000 |
| Core clinical model | Surgery duration, min | 1.004 | 1.003 | 1.006 | <0.001 | 0.001 | 0.000 |
| Core clinical model + imputed PFT | Age | 1.059 | 1.039 | 1.080 | <0.001 | 0.023 | 0.022 |
| Core clinical model + imputed PFT | Male sex | 1.069 | 0.812 | 1.407 | 0.634 | 0.009 | 0.008 |
| Core clinical model + imputed PFT | BMI | 0.958 | 0.931 | 0.985 | 0.003 | 0.037 | 0.036 |
| Core clinical model + imputed PFT | Current smoker | 1.717 | 1.231 | 2.395 | 0.002 | 0.023 | 0.022 |
| Core clinical model + imputed PFT | COPD | 2.556 | 1.859 | 3.517 | <0.001 | 0.031 | 0.030 |
| Core clinical model + imputed PFT | Heart failure | 2.336 | 1.639 | 3.329 | <0.001 | 0.026 | 0.025 |
| Core clinical model + imputed PFT | Diabetes mellitus | 1.050 | 0.784 | 1.406 | 0.745 | 0.023 | 0.022 |
| Core clinical model + imputed PFT | Chronic kidney disease | 1.563 | 1.139 | 2.146 | 0.006 | 0.016 | 0.015 |
| Core clinical model + imputed PFT | asa_class2 | 0.845 | 0.510 | 1.401 | 0.514 | 0.012 | 0.011 |
| Core clinical model + imputed PFT | ASA class III | 0.762 | 0.448 | 1.297 | 0.317 | 0.023 | 0.022 |
| Core clinical model + imputed PFT | ASA class IV | 1.074 | 0.600 | 1.922 | 0.810 | 0.017 | 0.016 |
| Core clinical model + imputed PFT | Functional dependence | 1.441 | 1.029 | 2.018 | 0.033 | 0.036 | 0.035 |
| Core clinical model + imputed PFT | Albumin, g/L | 0.837 | 0.811 | 0.864 | <0.001 | 0.024 | 0.023 |
| Core clinical model + imputed PFT | Emergency surgery | 1.923 | 1.382 | 2.676 | <0.001 | 0.046 | 0.045 |
| Core clinical model + imputed PFT | Upper abdominal site | 1.857 | 1.409 | 2.446 | <0.001 | 0.029 | 0.028 |
| Core clinical model + imputed PFT | Open approach | 1.475 | 1.112 | 1.956 | 0.007 | 0.045 | 0.044 |
| Core clinical model + imputed PFT | Surgery duration, min | 1.004 | 1.003 | 1.006 | <0.001 | 0.010 | 0.009 |
| Core clinical model + imputed PFT | FEV1, % predicted | 0.955 | 0.947 | 0.964 | <0.001 | 0.197 | 0.190 |
| Core clinical model + imputed PFT | FEV1/FVC ratio | 0.339 | 0.043 | 2.684 | 0.302 | 0.291 | 0.278 |

**Supplementary Table S4. Apparent discrimination and Brier score across MICE pulmonary function analyses.**

MICE estimates summarize apparent model performance across 10 imputed datasets and are sensitivity analyses rather than replacements for primary model validation.

| **Model** | **Auc Mean** | **Auc Sd** | **Auc Min** | **Auc Max** | **Brier Mean** | **Brier Sd** |
| --- | --- | --- | --- | --- | --- | --- |
| Core clinical model | 0.8426 | 0.0000 | 0.8426 | 0.8426 | 0.1022 | 0.0000 |
| Core clinical model + imputed PFT | 0.8769 | 0.0017 | 0.8748 | 0.8793 | 0.0896 | 0.0008 |

**Supplementary Table S5. Complete-case pulmonary function sensitivity analysis.**

This analysis compares the core clinical model with the core model plus pulmonary function variables among patients with complete pulmonary function data.

| **Analysis** | **Model** | **N** | **Events** | **Auc** | **Brier** |
| --- | --- | --- | --- | --- | --- |
| Complete-case PFT subset | Core clinical model | 1997 | 339 | 0.8400 | 0.1017 |
| Complete-case PFT subset | Core clinical model + PFT | 1997 | 339 | 0.8732 | 0.0898 |

**Supplementary Table S6. Common co-occurrence patterns among patients with PPC.**

The table shows common isolated or combined PPC component patterns among the 425 patients with PPC.

| **PPC component pattern** | **N** | **% of PPC cases** |
| --- | --- | --- |
| Pneumonia | 100 | 23.5 |
| Respiratory failure | 80 | 18.8 |
| Atelectasis | 70 | 16.5 |
| Pneumonia + Respiratory failure + Atelectasis | 28 | 6.6 |
| Prolonged mechanical ventilation >48 h | 20 | 4.7 |
| Unplanned reintubation | 18 | 4.2 |
| Pneumonia + Respiratory failure | 17 | 4.0 |
| ARDS | 16 | 3.8 |
| Pneumonia + Respiratory failure + Prolonged mechanical ventilation >48 h | 8 | 1.9 |
| Pneumonia + Atelectasis | 7 | 1.6 |
| Respiratory failure + Atelectasis | 7 | 1.6 |
| Pneumonia + Atelectasis + Prolonged mechanical ventilation >48 h | 5 | 1.2 |
| Pneumonia + Respiratory failure + Atelectasis + Prolonged mechanical ventilation >48 h | 5 | 1.2 |
| Pneumonia + Respiratory failure + Atelectasis + Unplanned reintubation | 5 | 1.2 |
| Pneumonia + Respiratory failure + Unplanned reintubation | 5 | 1.2 |
| Pneumonia + Prolonged mechanical ventilation >48 h | 4 | 0.9 |
| Pneumonia + Respiratory failure + ARDS | 4 | 0.9 |
| Respiratory failure + Atelectasis + ARDS | 3 | 0.7 |
| Atelectasis + ARDS | 2 | 0.5 |
| Pneumonia + Atelectasis + ARDS | 2 | 0.5 |

**Supplementary Table S7. Clinical outcomes by PPC severity.**

Major PPC was defined as any respiratory failure, ARDS, unplanned reintubation, or prolonged mechanical ventilation >48 h. Patients with pneumonia or atelectasis accompanied by any major component were classified into the major PPC group.

| **Outcome/component** | **No PPC (N=2,031)** | **Minor PPC (N=177)** | **Major PPC (N=248)** | **P value** |
| --- | --- | --- | --- | --- |
| Pneumonia | 0 (0%) | 107 (60%) | 91 (37%) | <0.001 |
| Respiratory failure | 0 (0%) | 0 (0%) | 176 (71%) | <0.001 |
| Atelectasis | 0 (0%) | 77 (44%) | 65 (26%) | <0.001 |
| Prolonged mechanical ventilation >48 h | 0 (0%) | 0 (0%) | 52 (21%) | <0.001 |
| Unplanned reintubation | 0 (0%) | 0 (0%) | 41 (17%) | <0.001 |
| ARDS | 0 (0%) | 0 (0%) | 34 (14%) | <0.001 |
| Hospital length of stay, days | 7.0 (5.0, 10.0) | 18.0 (12.0, 26.0) | 17.5 (14.0, 24.0) | <0.001 |
| ICU admission | 256 (13%) | 81 (46%) | 120 (48%) | <0.001 |
| Postoperative mechanical ventilation | 87 (4.3%) | 57 (32%) | 99 (40%) | <0.001 |
| 30-day mortality | 56 (2.8%) | 27 (15%) | 27 (11%) | <0.001 |
| 90-day mortality | 68 (3.3%) | 36 (20%) | 42 (17%) | <0.001 |
| 30-day hospital readmission | 187 (9.2%) | 40 (23%) | 58 (23%) | <0.001 |
| Total hospital cost, CNY | 23,490.0 (14,960.0, 39,010.0) | 68,750.0 (46,060.0, 100,070.0) | 70,300.0 (48,875.0, 100,860.0) | <0.001 |

**Supplementary Table S8. XGBoost performance by cohort split.**

Performance was evaluated across training, internal-validation, independent-test, and external-validation datasets.

| **Cohort** | **PPC, n/N (%)** | **AUC-ROC (95% CI)** |
| --- | --- | --- |
| Training | 297/1719 (17.3%) | 0.833 (0.812-0.854) |
| Internal validation | 64/369 (17.3%) | 0.820 (0.774-0.866) |
| Independent test | 64/368 (17.4%) | 0.856 (0.811-0.900) |
| External validation | 105/542 (19.4%) | 0.821 (0.781-0.861) |

**Supplementary Table S9. Threshold metrics for XGBoost by cohort split.**

Sensitivity, specificity, PPV, NPV, accuracy, Brier score, and confusion-matrix counts are shown at the 20% risk threshold.

| **Cohort** | **Threshold** | **Sensitivity** | **Specificity** | **PPV** | **NPV** | **Accuracy** | **Brier** | **TP** | **TN** | **FP** | **FN** |
| --- | --- | --- | --- | --- | --- | --- | --- | --- | --- | --- | --- |
| Training | 0.2 | 0.791 | 0.662 | 0.328 | 0.938 | 0.684 | 0.113 | 235 | 941 | 481 | 62 |
| Internal validation | 0.2 | 0.750 | 0.675 | 0.327 | 0.928 | 0.688 | 0.117 | 48 | 206 | 99 | 16 |
| Independent test | 0.2 | 0.875 | 0.609 | 0.320 | 0.959 | 0.655 | 0.107 | 56 | 185 | 119 | 8 |
| External validation | 0.2 | 0.790 | 0.657 | 0.356 | 0.929 | 0.683 | 0.122 | 83 | 287 | 150 | 22 |

**Supplementary Table S10. Discrimination of XGBoost and comparator scores.**

ARISCAT and NSQIP analyses were restricted to patients with complete score components. External comparator-score analyses were exploratory.

| **Cohort** | **Score/model** | **N** | **Events** | **AUC-ROC (95% CI)** |
| --- | --- | --- | --- | --- |
| Development/internal | XGBoost | 2456 | 425 | 0.833 (0.816-0.851) |
| Development/internal | LightGBM | 2456 | 425 | 0.818 (0.799-0.837) |
| Development/internal | Logistic regression | 2456 | 425 | 0.693 (0.665-0.721) |
| Development/internal ARISCAT subset | ARISCAT | 1247 | 218 | 0.736 (0.700-0.773) |
| Development/internal NSQIP subset | NSQIP | 1832 | 313 | 0.679 (0.644-0.714) |
| External validation | XGBoost | 542 | 105 | 0.821 (0.781-0.861) |
| External validation | LightGBM | 542 | 105 | 0.805 (0.761-0.848) |
| External validation | Logistic regression | 542 | 105 | 0.699 (0.642-0.755) |
| External validation ARISCAT subset | ARISCAT | 282 | 52 | 0.707 (0.627-0.788) |
| External validation NSQIP subset | NSQIP | 410 | 76 | 0.724 (0.660-0.787) |

**Supplementary Table S11. Temporal model performance by study year.**

Annual performance was evaluated to explore temporal stability over the 2018-2022 study period.

| **Year** | **N** | **PPC events** | **PPC rate, %** | **AUC-ROC (95% CI)** | **Brier** |
| --- | --- | --- | --- | --- | --- |
| 2018 | 510 | 97 | 19.0 | 0.797 (0.755-0.839) | 0.127 |
| 2019 | 500 | 91 | 18.2 | 0.851 (0.816-0.887) | 0.113 |
| 2020 | 485 | 84 | 17.3 | 0.819 (0.777-0.860) | 0.116 |
| 2021 | 480 | 77 | 16.0 | 0.865 (0.828-0.902) | 0.103 |
| 2022 | 481 | 76 | 15.8 | 0.847 (0.807-0.886) | 0.103 |

**Supplementary Table S12. Calendar-year and COVID-era sensitivity models.**

Intercept terms were omitted because they are not clinically interpretable. OR, odds ratio; CI, confidence interval.

| **Model** | **Term** | **OR (95% CI)** | **P value** |
| --- | --- | --- | --- |
| Calendar-year trend | study_year | 0.94 (0.87-1.01) | 0.110 |
| COVID-period comparison | covid_periodCOVID-era period (2020-2022) | 0.86 (0.69-1.06) | 0.152 |

**Supplementary Table S13. Descriptive length-of-stay and cost comparisons by PPC severity.**

This is a descriptive burden analysis only and should not be interpreted as a cost-effectiveness analysis.

| **Ppc Severity** | **N** | **Median Cost** | **P25 Cost** | **P75 Cost** | **Median Los** | **P25 Los** | **P75 Los** |
| --- | --- | --- | --- | --- | --- | --- | --- |
| No PPC | 2031 | 23490 | 14965.0 | 38985 | 7.0 | 5 | 10 |
| Minor PPC | 177 | 68750 | 46060.0 | 100070 | 18.0 | 12 | 26 |
| Major PPC | 248 | 70300 | 49052.5 | 100820 | 17.5 | 14 | 24 |

# Supplementary figure


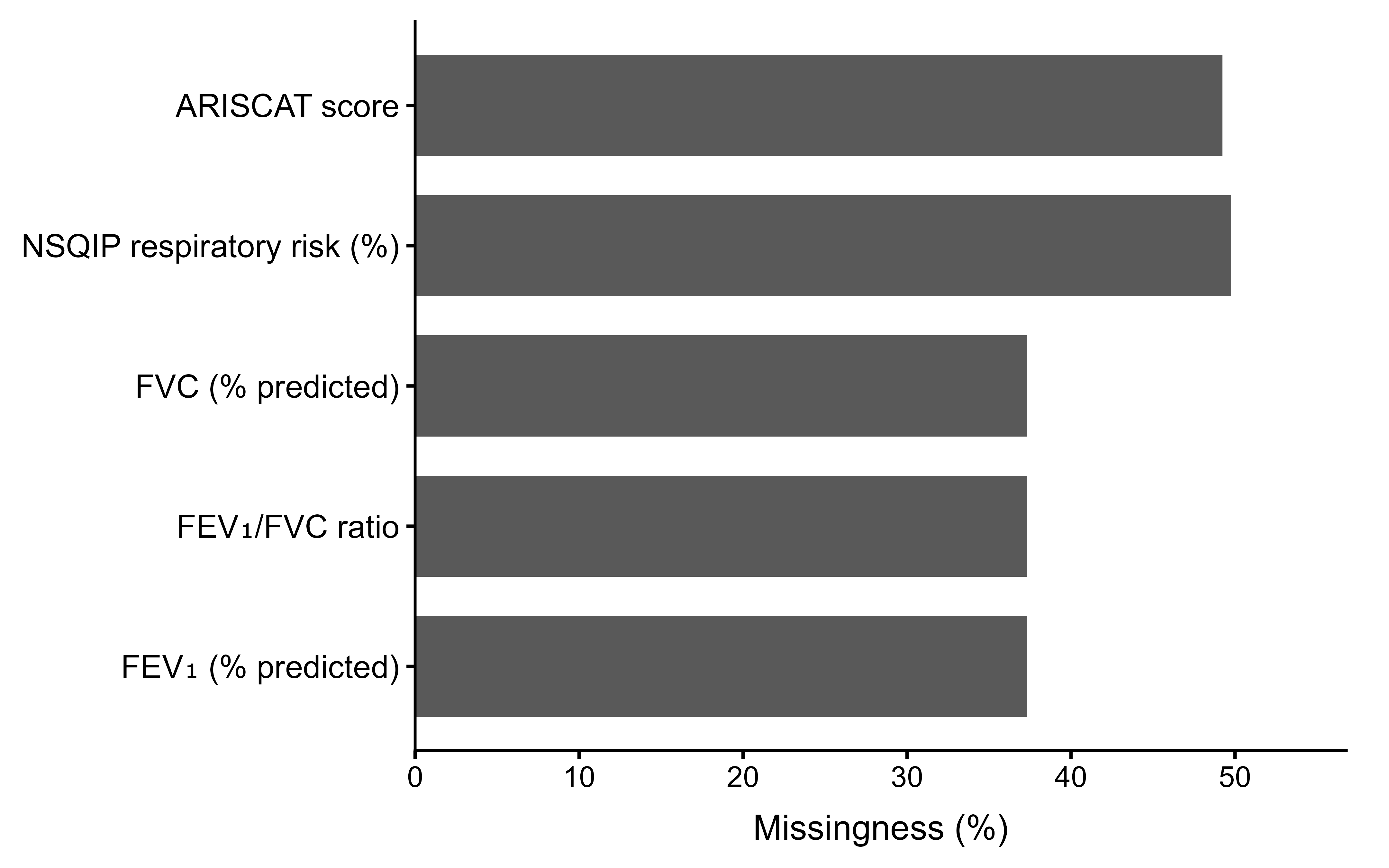


Supplementary Figure S1. Missingness of candidate predictors in the development/internal cohort. The horizontal bar chart shows percentage missingness for variables with meaningful incomplete data. Pulmonary function variables had moderate missingness, whereas ARISCAT and NSQIP were treated as comparator scores rather than primary candidate predictors.


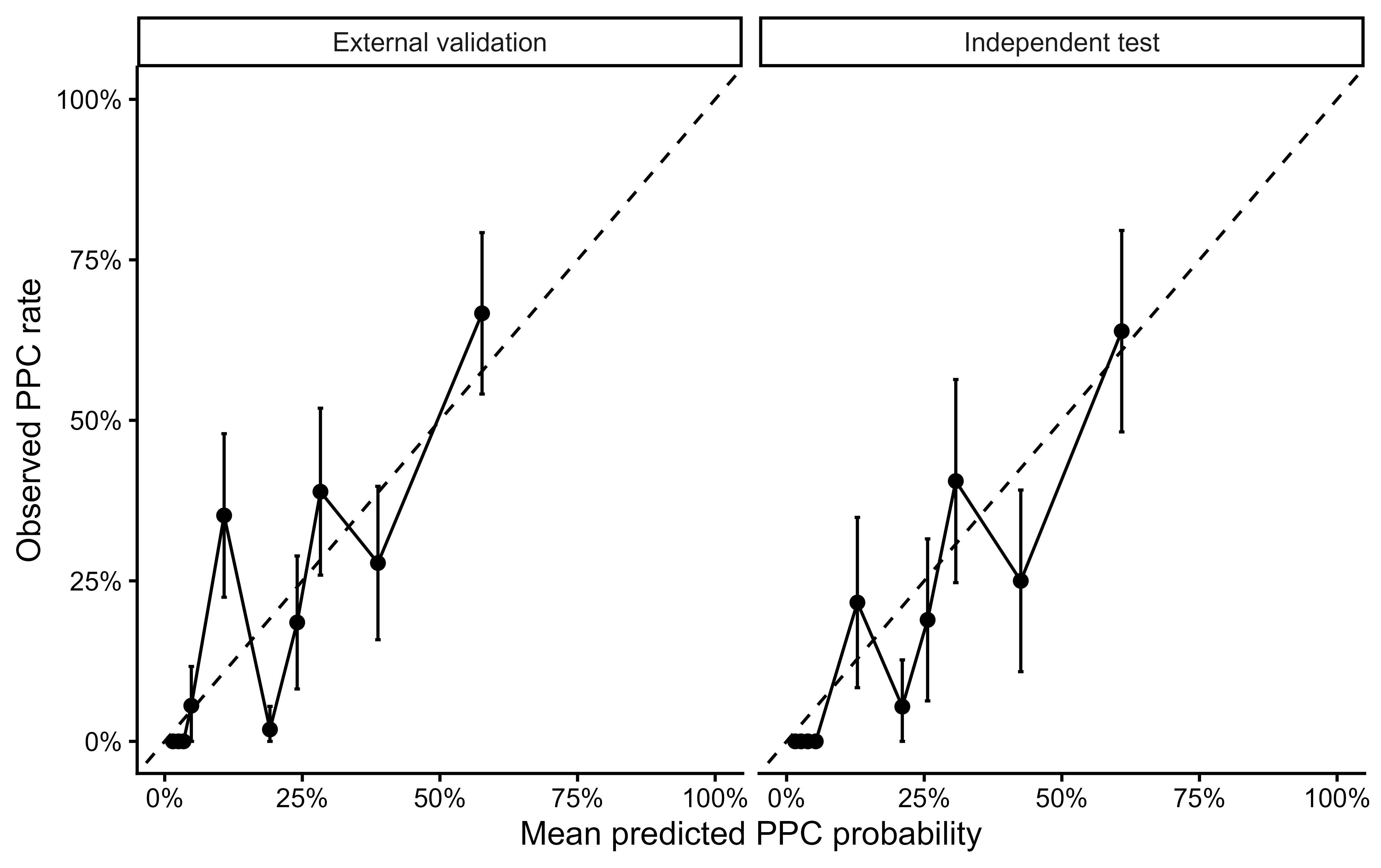


Supplementary Figure S2. Calibration of XGBoost in the independent test and external-validation cohorts. Points show observed PPC risk by decile of predicted risk; the diagonal line indicates perfect calibration. Decile-level variability should be interpreted in light of the number of events within each stratum.


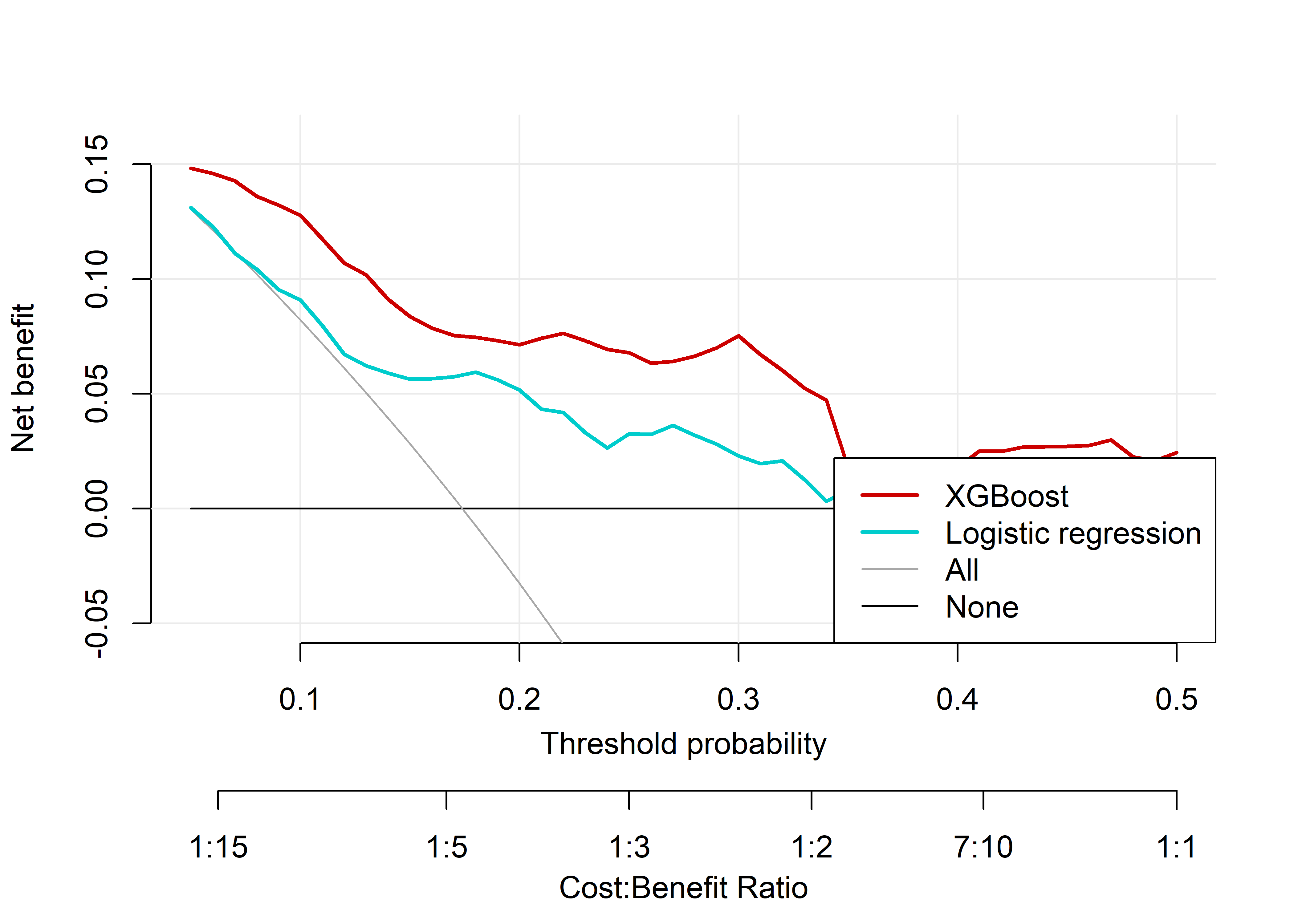


Supplementary Figure S3. Decision curve analysis comparing XGBoost with logistic regression in the independent test set. Net benefit is plotted across threshold probabilities from 5% to 50%. The analysis supports clinical utility across a range of plausible perioperative decision thresholds.


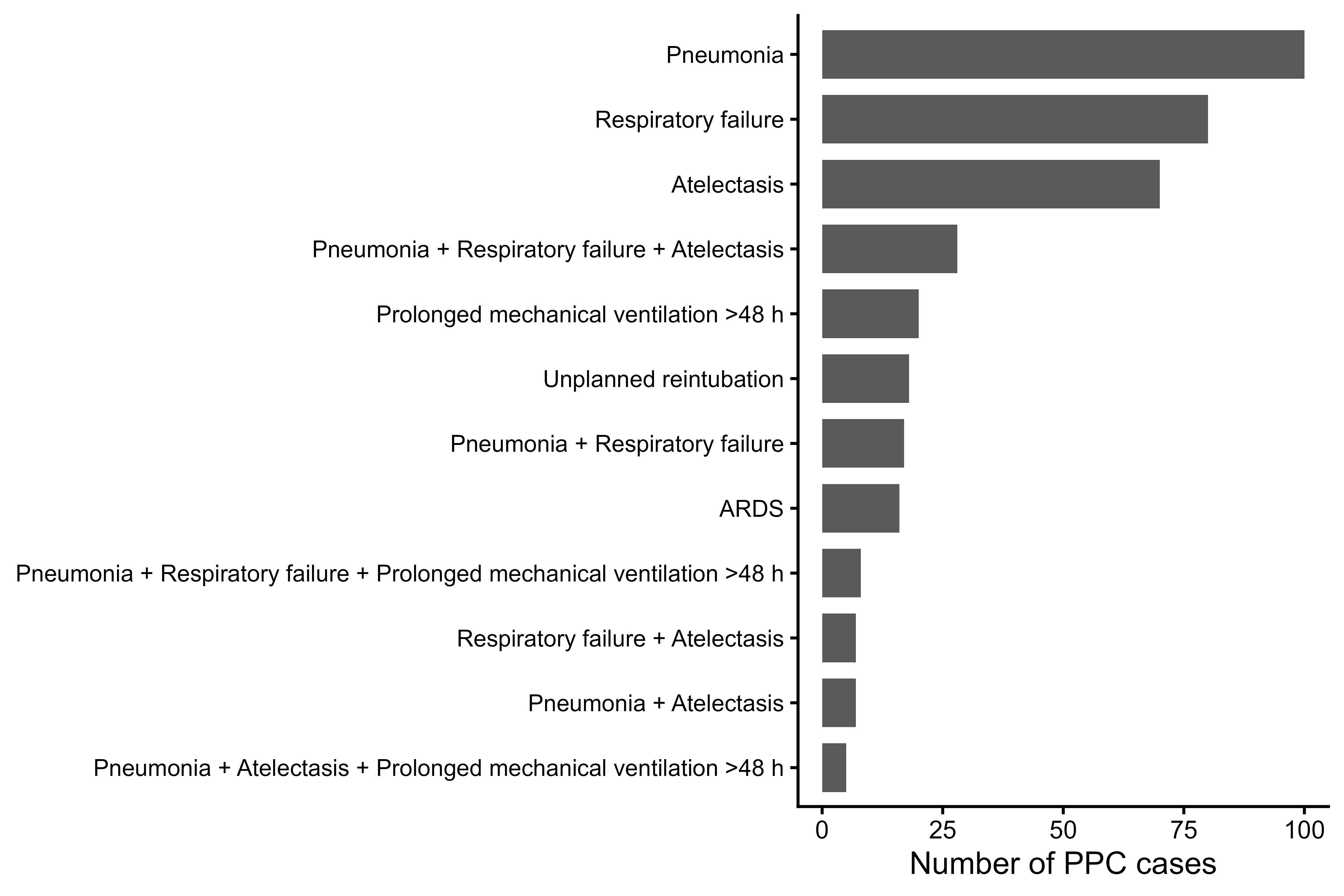


Supplementary Figure S4. Common co-occurrence patterns among PPC components. Bars summarize isolated and combined pulmonary complication patterns among patients who developed at least one PPC.


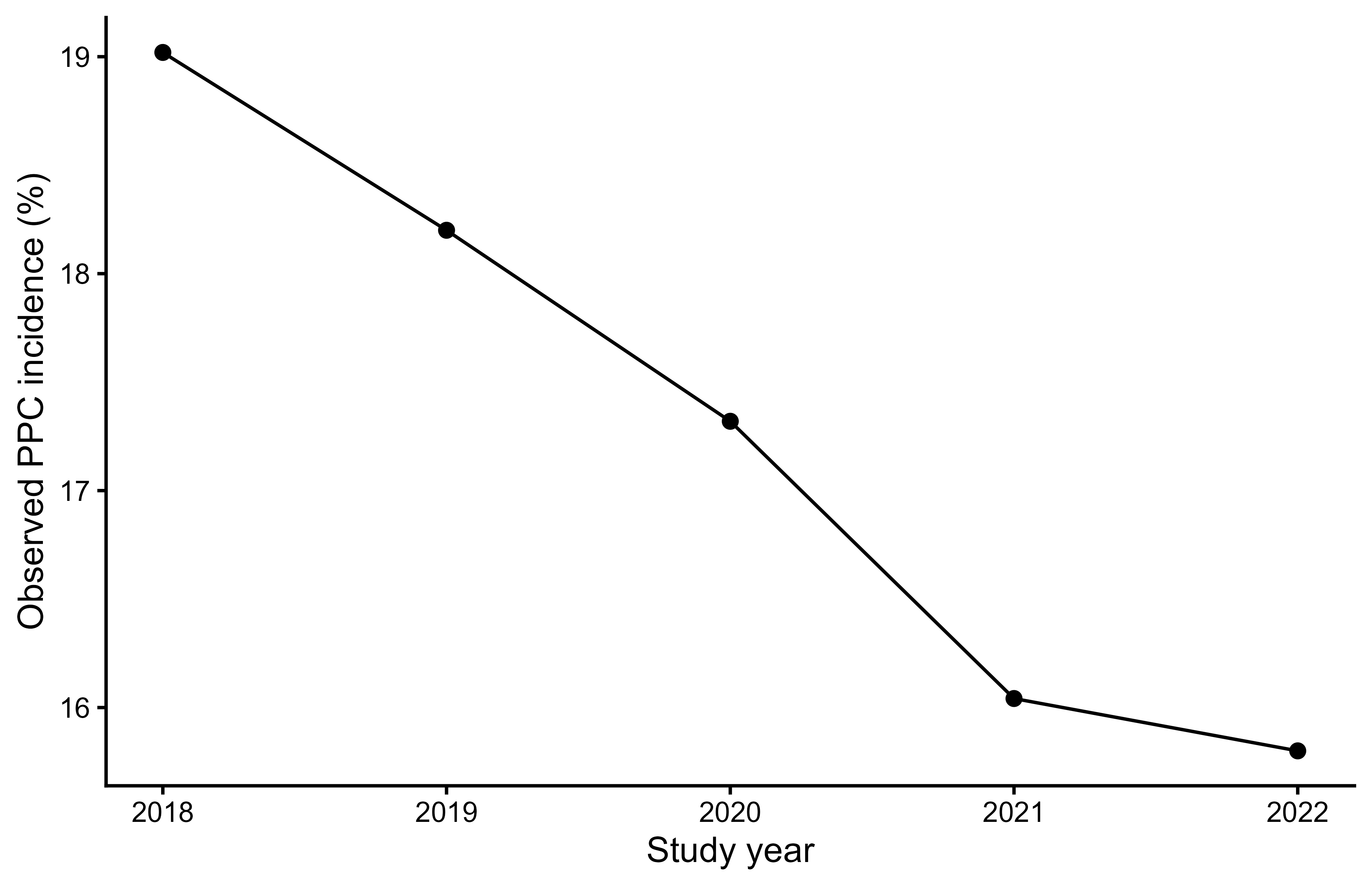


Supplementary Figure S5. Annual PPC incidence across the study period. Annual event rates are shown to contextualize temporal trends and COVID-era sensitivity analyses.
